# Shifting the Overton Window: enhancing therapeutic outcomes for Māori experiencing Ngā Māuiui kai (eating disorders) through the integration of traditional Māori and Western healing systems in Aotearoa New Zealand

**DOI:** 10.3389/fpsyt.2026.1795945

**Published:** 2026-05-15

**Authors:** Mau te Rangimarie Clark, Riki Tumohe Clark, Jenny Jordan, Richard Porter, Suzanne Pitama

**Affiliations:** 1 Department of Psychological Medicine, University of Otago, Christchurch, New Zealand; 2 Te Haa o Io, Rongoā Māori & Creative Arts Therapy, Christchurch, New Zealand; 3 Department of Māori Indigenous Health Innovation, University of Otago, Christchurch, New Zealand

**Keywords:** cultural adaptation, eating disorders, Indigenous mental health, psychotherapy, Rongoā Māori

## Abstract

Ngā māuiui kai (eating disorders) are understood within a Māori worldview as a manifestation of imbalance across hinengaro (mind), tinana (body), wairua (spirit) and whānau (family or support system). Prevailing Western treatment, which prioritise biopsychosocial interventions, do not address the cultural and spiritual determinants of health for Māori, creating a gap in culturally responsive care for Māori. This gap is rooted in and sustained by the enduring impacts of colonisation, which marginalised mātauranga Māori (Māori knowledge) and disrupted traditional healing systems. This paper proposes that Specialist Supportive Clinical Management, a flexible, patient-centred psychotherapy, provides a unique point of alignment for the integration of Rongoā Māori, the traditional Māori healing system. Rongoā Māori encompasses modalities such as rongoā rākau (herbal remedies), mirimiri/romiromi (physical therapies), puku kōrero (talk therapy), and karakia (incantations/prayer) and offers a framework embedded within Māori knowledge systems. We argue that rongoā-informed Specialist Supportive Clinical Management, where puku kōrero aligns with Specialist Supportive Clinical Management patient-led dialogue, can synergistically support biopsychosocial goals while ensuring a holistic, culturally grounded approach. This conceptual analysis proposes a culturally adapted intervention to enhance therapeutic engagement, improve health outcomes, and honour the treatment aspirations of Māori by addressing the whole person within their cultural context.

## Introduction

Ngā māuiui kai or eating disorders (ED) are a significant global health issue, including for Indigenous peoples and ethnic minority groups (1). Studies show prevalence rates of Indigenous populations are equal to or higher than those of settler-colonial or White-majority counterparts (2, 3). In Aotearoa New Zealand (NZ), inequities for Māori experiencing ED has been formally recognised in the governments refreshed ‘New Zealand Eating Issues and Eating Disorders Strategy (NZEIEDS).’ (4) That document not only acknowledges the growing ED literature, but it also validates Māori conceptualisations of ED by incorporating the newly created te reo Māori (Māori language) glossary (5). Crucially, the NZEIEDS calls for treatment responsive to NZ’s diversity and acknowledges the need for future investment in kaupapa Māori (Māori approach, Māori agenda) primary healthcare services to improve equitable access to care.

Kaupapa Māori primary healthcare services respond to the needs of their communities and operate through tikanga (cultural protocols) and mātauranga Māori (Māori knowledge) (6). Rolleston et al. (2020) (7) argues that recognising mātauranga Māori-based health interventions are a necessary component for achieving health equity in NZ. To our knowledge, no kaupapa Māori services have been operationalised as spaces through which treatment for ED are delivered. Two central hypotheses are considered: first, that dominant social narratives incorrectly frame EDs as a disorder affecting skinny, white, affluent, girls (SWAG) (8); and second, that mātauranga Māori has been excluded from mainstream ED conceptualisations and clinical spaces (5). However, the government’s recent recognition of Māori conceptualisations of EDs and Eating Issues give rise to questions concerning culturally responsive treatment modalities (4).

This paper argues for a necessary shift in the Overton Window^1^ (9), the spectrum of politically acceptable ideas in ED treatment, moving from a dominant Western biopsychosocial framework (a genuine advance beyond purely biomedical models) toward biopsychosociocultural enactment, that genuinely embraces Indigenous healing knowledges as a valid and essential component of effective treatment.

## Positionality: Kaupapa Māori methodologies

This manuscript is positioned within Kaupapa Māori Methodologies, a Māori-centred approach that prioritises Māori worldviews, knowledge systems, and agendas. The development of Kaupapa Māori theory and methodologies is, however, the result of a collective effort by many Māori scholars, including Linda and Graham Smith, and Leonie Pihama, among others (10–12). Since then, a substantial body of literature detailing kaupapa Māori methodologies has been collated by Indigenous and non-Indigenous scholars and clinicians internationally (13–15). Central to Kaupapa Māori Theory is the validation of Māori knowledge systems as legitimate and authoritative, particularly in critiquing colonial structures and their ongoing effects within health and research systems. Accordingly, our search strategy was designed to be responsive to the specific epistemological and material constraints of the existing literature on Rongoā Māori, recognising that mainstream health research has historically marginalised or excluded Indigenous healing knowledges.

We employed pragmatic, iterative search (16) using google scholar and google search with terms including “Rongoā Māori” and “rongoa.” We sampled this with searching of reference lists (snowball sampling). Rather than applying a single hierarchy of evidence that would privilege randomised controlled trials (of which very few exist for Rongoā Māori), we deliberately sought diverse source types; peer-reviewed articles, government documents, kaupapa Māori (Māori approach, Māori agenda) service reports, and publications from Māori research centres. We did not apply a formal quality appraisal as this is a conceptual synthesis, but we note where evidence types differ in the body of the manuscript.

## The current landscape: eating disorders and Māori communities

Recent research revealed significant disparities in treatment of ED for Māori. Analysis of national mental health service use data (2009 to 2016) revealed that Māori only comprised 7% of those receiving treatment who had an eating disorder diagnosis (3), half of that expected given the proportion of Māori in the population and the at least comparable ED rates in Māori compared to non-Māori reported in an epidemiology study (17). That epidemiology study reported lifetime prevalences for māuiui whakatiki (AN) 0.7% and pukuruaki (BN) 2.4% for Māori, whilst non-Māori rates were 0.6% and 1.3% respectively (17).

In the service utilisation study noted above, there were fewer than expected ED being treated for Māori with māuiui whakatiki or Anorexia Nervosa (AN), but higher than expected rates with pukuruaki or Bulimia Nervosa (BN), and greater comorbidities, including depression, anxiety, and substance use and higher utilisation of crisis and addiction services (3). Other studies suggest that this under-representation of Māori in ED treatment is likely to be due to a range of barriers to treatment related to clinician bias, inconsistent use of assessment methods, inaccessible service locations, limited number of inpatient beds and shame and stigma (18). This disparity reflects a system that is ill-equipped to recognise, assess, and treat Māori.

For Māori, this is compounded by unique structural determinants rooted in the intergenerational impacts of colonisation. Quantitative data reveal a stark pattern that the experience of EDs is distinctly shaped by deprivation: in the service utilisation study, Māori receiving treatment were most likely to be residing in the most socioeconomically deprived areas, whereas non-Māori were more likely to be in the least deprived quintile (3). This pattern confirms the “affluent” stereotype for the non-Māori population while inverting it for Māori, directly highlighting non-Māori privilege within the healthcare system. Non-Māori as the key benefactors of settler-colonial structures, maintain greater access to the social determinants of health including specialist care. Furthermore, qualitative findings indicate that Māori themselves frequently attribute the development of EDs to cumulative and intersecting adversities starting from childhood (19). These include the imposition of Western body image ideals, experiences of trauma (including sexual, physical, and emotional violence) (20), and profound grief (from death or whānau divorce) (19). These factors, such as trauma and poverty are recognised as bearing a disproportionate burden on Māori communities, thereby constituting critical pathways to be considered in the aetiology of EDs.

This critique of incongruent assessment methods finds a parallel in contemporary developments within Western psychiatry itself. In late 2025, the Diagnostic Statistical Manual (DSM) Steering Committee proposed a significant revision to the severity specifiers for AN, BN, and māuiui kaihoro, or Binge Eating Disorder (BED). The proposed change seeks to “emphasise the importance of symptom severity, functional impairment, and illness-related medical complications rather than relying on” BMI or behavioural frequency alone (21). This shift acknowledges that purely weight-centric metrics have been insufficient for many patient populations including Māori and other Indigenous populations (2).

From a Te Ao Māori (Māori worldview) perspective, even the revised reframing remains incomplete. Where the DSM conceptualises impairment individualistically and symptomatically, a Māori worldview would also attend to the rupture of whakapapa (genealogical) connections, the diminishment of wairua (spirit), or the collective burden on whānau mana (vitality pertaining to family/support network). These are not merely different measures of the same construct; they represent a different understanding of what ‘impairment’ and ‘severity’ mean.

Rather than rejecting the DSM framework entirely, we suggest that it can be usefully supplemented by a Māori worldview. Where the DSM provides a formal diagnostic structure, important for clinical communication and services access, the newly established ED glossary offers a complementary framework for case conceptualisation: understanding the meaning of an ED within whakapapa (genealogical connections), whānau (support system) and wairua (spirit). In this spirit, we see complementarity between diagnostic systems, not necessarily opposition.

## Ngā māuiui kai: eating disorders

The term ngā māuiui kai was developed in 2023 by Te Tira Wānanga Māuiui Kai (the Māori Eating Disorders Network), a collective of Māori lived-experience experts, whānau (support network), carers, clinicians, policy makers, cultural consultants, linguists, and researchers (5). Their priority was to establish a shared, culturally authentic language for disordered eating, resulting in a transformative glossary that conceptualises these conditions from a Māori worldview (5). Central to this framework is understanding ED not as a fixed illness, but as a state of imbalance affecting tinana (physical), wairua (spiritual), hinengaro (mental), and whānau (family, support network) (5). Within this framework, health is an all-encompassing concept of keeping a healthy mind, body, soul, and social connections rather than just the absence of sickness. Here, māuiui signifies being unwell, out of sorts, or out of balance, while kai translates to food. Thus, ED represents a recoverable imbalance in one’s relationship with food, and that disordered eating is viewed as a signal of a deeper imbalance rather than the core issue itself. This nuanced perspective is further illustrated in terms like pukuruaki for BN. In te ao Māori (the Māori worldview), the puku (stomach) is where emotions are held; ruaki means to expel. The term thus conceptually frames the behaviour as an attempt to manage or expel stored emotions.

The reclamation of te reo Māori (Māori language) through this glossary is a vital act of self-determination, restoring Māori conceptualisation of health and wellbeing. The Māori language, once threatened with extinction, has seen revitalisation since the 1970s through relentless community activism (22). Using these terms is therefore both a clinical and political act. It directly challenges the dominance of Western psychological constructs and, in doing so, actively shifts the Overton Window of acceptable treatment discourse. By affirming the validity of Indigenous knowledge as foundational to clinical practice, this reclamation expands the spectrum of politically and therapeutically legitimate ideas, moving Māori paradigms from the margins to the centre of effective care for ED.

## Indigenous rights, and Te Tiriti o Waitangi

The call for culturally grounded ED treatment is supported by a robust international framework for Indigenous rights. Article 24 of the united Nations Declaration on the Rights of Indigenous peoples (UNDRIP) explicitly states this (23). Adopted in 2007 after decades of advocacy, UNDRIP recognises the historical and ongoing impacts of colonisation affirming Indigenous rights to self-determination, health, language, religious customs, culture and identity, rights to education, and rights to economic development (24). Articles 24, 18, 2, 3 and 11 address health and well-being, participation in decision-making, discrimination, and cultural preservation and self-determination.

However, the justification for Indigenous rights remains fundamentally moral, arising in direct response to systematic wrongdoings that continue to marginalise Indigenous peoples. While UNDRIP provides crucial security at the global level, its enactment depends on national political will (25). Consequently, advocates maintain that ongoing international scrutiny and pressure are vital to compel government compliance and realise these rights in practice (26).

In NZ, settler colonialism was enabled through a signed agreement between Māori chiefs, representing tribes across the country, and the British-Crown (27). That agreement, known as Te Tiriti o Waitangi, is often described as the nation’s founding document. However, interpretations and honouring of Te Tiriti, have been contested by successive governments since its signing. Discrepancies between the English text and the Reo Māori (Māori language) text have been used as a pretext to challenge the documents very legitimacy (28). Aligning with broader Indigenous rights movements in the 1980s, the discourse moved towards reframing Te Tiriti as a living partnership.

That shift led to the establishment of key principles, partnership, participation, and protection, to reconcile differences between the Māori and English texts and apply the covenant to contemporary circumstances (29, 30). These principles are now central to efforts to redress past injustices, mandating that the NZ government work in partnership with Māori. However, the interpretation and implementation of these principles remain vulnerable to shifts in political priorities across successive governments.

## Current treatment modalities: a place of exclusion for Māori

Consideration of culturally responsive treatment for ED aligns with international critiques of conventional ED support (31). Scholars advocate for an intersectional approach, arguing that prevailing methods often centre on ‘White, hyper-medicalised and restrictive’ practices that can conflict with the values and belief systems of diverse global populations (32). Current first-line treatments for māuiui whakatiki (AN), pukuruaki (BN), and māuiui kaihoro (BED) are primarily psychological, such as family therapy for youth, cognitive behavioural therapy, or pharmacological, or a combination thereof (33). However, even those gold standard evidence-based therapies often yield inadequate outcomes, particularly for AN, a limitation frequently attributed to an overly narrow, weight-centric focus (33).

The call for holistic outcome measures arises, in part, from critique of Western-informed treatment paradigms (34). Phillipou et al. (34) identify the root issue as an entrenched body-mind dichotomy that has historically characterised much of Western medicine. That division of the body-mind manifests clinically as siloed care, wherein responsibilities are split. For instance, a general practitioner may focus solely on weight restoration while a therapist addresses cognitive patterns, with little integration of the two. Whilst we agree with the need for a multidisciplinary approach to ED, Philippou et al. asserts that for measures to be truly holistic, outcome measures must be designed to address the underlying causal factors concurrently with eating behaviours themselves. The advent of specialty services has meant that those services often will not treat comorbidity such as substance use, trauma or personality disorder issues.

The concept of holistic health from a Te Ao Māori (Māori worldview) explicitly incorporates spiritual and relational dimensions of wellbeing. This is reflected in a recent systematic review of Māori health models, which identified four central themes: 1) dimensions of holistic health and wellbeing, 2) whanaungatanga (connectedness), 3) whakawhanaungatanga (building relationships), and 4) socio-political health context (3). Within this framework, rongoā (remedy), a traditional Māori healing system emerges as a practice that could significantly enhance Western treatment modalities. When treatment fails to align with the cultural values, belief systems, and lived realities of the populations it serves, engagement declines, outcomes worsen, and existing health inequities are exacerbated. For Māori with ED, the absence of culturally grounded treatment pathways perpetuates a cycle of underutilisations, widening the gap between Māori and non-Māori health outcomes.

Therefore, a culturally responsive treatment model is necessary to authentically embed a holistic, Māori-centred worldview while addressing the self-perceived causal factors for ED. ‘Specialist Supportive Clinical Management’ (SSCM), an evidence-based psychotherapy developed in Aotearoa (35) presents a promising candidate for such adaptation.

## Specialist supportive clinical management: an evidence-based therapy

### What is specialist supportive clinical management?

While there are several established evidence-based interventions for ED, they are not universally effective and often result in only short-term improvements (36). Consequently, progress in the field remains slow, as even those who respond to initial treatment frequently experience high rates of relapse (36). The difficulty in establishing a “gold standard” of care is particularly evident in adult treatment, where Specialist Supportive Clinical Management (SSCM) has been found to be as effective as more specialised complex psychological therapies.

Specialist Supportive Clinical Management was originally constructed by drawing on well-established best practice principles of clinical management for AN used in ED treatment settings, and on long-established widely used supportive therapy principles and strategies, many of which are common to other counselling or psychotherapy approaches (37). As such, both components are well-established and familiar to many therapists. It has been argued that SSCM offers necessary treatment components, whereas other more complex contemporary therapies also include additional foci and strategies related to their overarching models (37).

The pre-requisites for using SSCM include a basic knowledge about ED and counselling skills. Specialist Supportive Clinical Management serves as a supportive therapy combining two central components; 1. clinical management (practical and symptom focussed strategies), 2. supportive psychotherapy. Specialist Supportive Clinical Management is pragmatic, atheoretical treatment client centred, strengths focussed therapy which allows room for patient perspectives of causation of their ED.

The clinical management component is a key prescriptive feature focussing on psychoeducation, nutritional advice, emphasis on the resumption of normal eating patterns, and restoration of weight (for those who are underweight). Whilst there are critiques about hyper-focus on weight, a recent study has found that clinicians and patients agreed that therapeutic benefit to discussing expected body weight, however, this was only achieved when timing, framing and individual patient-needs were considered (38).

With SSCM, the core message conveyed to patients is that improving their nutrition will result in the improvement of their physical and emotional health. The supportive psychotherapy component of SSCM is dictated by the patient who can focus on any aspect of their life whether it is related to the ED or presenting concern, be it whānau (family, support network), identity, or trauma. Specialist Supportive Clinical Management emphasises the therapeutic relationship through providing care, support, warmth, acceptance to enhance engagement in treatment. The therapist’s task is to listen and assist the person to find ways of dealing with their life issues, drawing on their strengths, but providing advice if that is wanted.

### What evidence is available for SSCM?

Against hypothesis, in the original trial SSCM outperformed Cognitive Behavioural Therapy (CBT) and Interpersonal Therapy (35). At follow-up though, the therapies were comparable (39). In subsequent trials, SSCM has been used as an active control and found to be comparable on primary ED outcomes in those with AN (strict and broadly defined) in six randomised controlled trials (RCT) against Mantra (40–42), CBT-E (42), against mentalisation for ED (non-underweight) with borderline personality disorder (43) or ED without borderline personality disorder (44). SSCM and CBT were adapted and found to be comparable for severe and enduring AN (45). In an open trial effectiveness study in a clinical setting with those with AN (strict and broadly defined), SSCM was found to be effective with comparable effect sizes achieved in research RCTs (46). Treatment credibility and acceptability have generally been reported to be comparable between SSCM and other therapies trialled (35, 42) with one exception, where those in the MANTRA arm submitted more positive feedback (41). The acceptability of SSCM for Māori is not yet known.

## Rongoā Māori: a traditional healing system

### What is rongoā Māori?

Rongoā Māori is more than physical therapies such as mirimiri (massage) or pani (ointment); it is described as a holistic system of healing deeply rooted in Māori cultural traditions. Mark et al. (2022) (47) frames Rongoā Māori as a biosphere, comprising a complex and comprehensive environment where healing practices are inextricably intertwined with intimate knowledge of the physical environment, including air, water, and the ground. A recent qualitative study comprised of 49 rongoā practitioners and patients identified four themes when conceptualising the holistic nature and meaning of Rongoā Māori- the themes included; land as an intrinsic part of identity; land as a site and source of healing; reciprocity of the healing relationship; and the importance of kaitiakitanga/conservation to Rongoā Māori (48).

**Figure 1:**
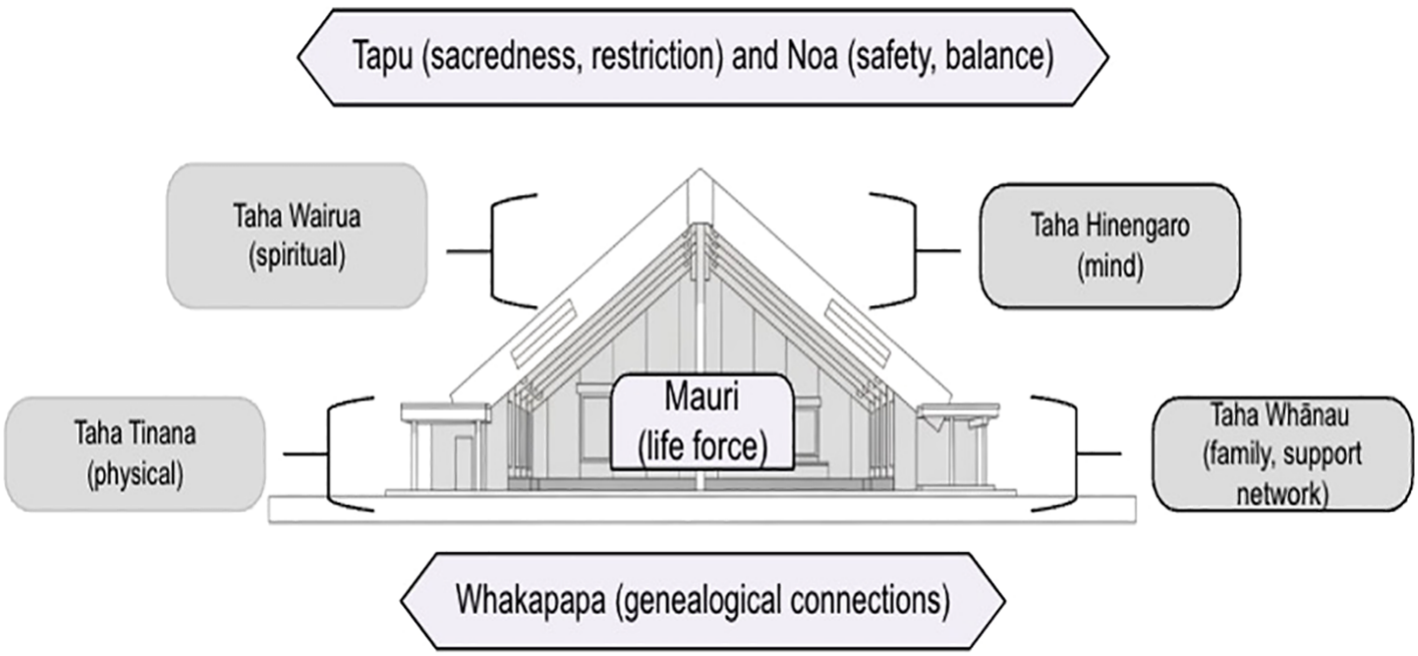
Te Whare Tapa Wha (The Four-Sided House). This model uses the powerful analogy of a wharenui (Māori meeting house) to represent a person’s holistic health and identity. The concepts of tapu (sacredness, restriction), noa (safety, balance), mauri (life force), and whakapapa (genealogical connections) have been added to this illustration.

Rongoā Māori encompasses Māori health models like Te Whare Tapa Wha (the four-sided house) (49), which identifies four essential dimensions of holistic wellbeing: wairua (spirit), hinengaro (mind), tinana (body) and whānau (family or support network). Rongoā Māori is governed by key Māori concepts, including mauri (the vital life force inherent in all beings and places), tapu (sacredness, restriction), and noa (the state of safety and balance achieved when tapu is properly managed) (50, 51). Sickness is understood as a disruption of mauri and an imbalance among these dimensions. Therefore, healing seeks to restore ora (wellbeing) by diagnosing and addressing disturbances across wairua (spirit), hinengaro (mind), tinana (body), and whānau (family, support network), and through realigning balance the individual’s whakapapa (genealogical) connections to the wider environment. **Figure 1** provides a visual illustration of the Te Whare Tapa Wha model.

Rongoā practitioners draw from a diverse toolkit that can include spiritual insight, cleansing rituals and physical therapies. The specific approach is deeply personal, shaped by the rongoā practitioner’s own lineage and relationship with their environment (52). Rongoā modalities are diverse and may include karakia (prayer, incantation) to address wairua (spirit), mirimiri (body work) to ease physical blockages, or rongoā rakau (plant remedy), and matakite (spiritual insight) to restore ora (Wellbeing, life) to a person’s entire world, thus their physical body, their mind, their family connections and their spiritual core (53, 54).

Historically, this knowledge was held by specialists called tohunga (expert) who were trained in whare wānanga (formal learning institutions, schools of sacred learning) (Jones, 2000b) (55). Tohunga use multi-step diagnostic process involving initial impressions, kōrero (discussion), and physical or spiritual examinations to detect underlying issues. Colonisation, including laws that banned their practice such as the Tohunga Suppression Act (1907) (56), violently disrupted this system, severing vital links in the chain of knowledge and eroded Māori trust in their own healing systems. The consequences of this disruption continue to affect Māori health today. Contemporary efforts to revitalise rongoā are acts of cultural reaffirmation. Modern whare wānanga now teach mātauranga Māori, including rongoā, alongside other arts like whakairo (carving).

This revival is crucial, as rongoā offers a paradigm of care that aligns with the need for holistic ED treatment for Māori. Its focus on systemic balance, spiritual wellbeing, and interconnectedness provides the very framework needed to address the imbalances signalled by ED in a culturally response way.

### What evidence is available for rongoā Māori?

The research literature on Rongoā Māori is significantly smaller than that for SSCM, and consists predominantly of qualitative studies, experiential reports, usage data, and biological plausibility arguments (48, 50, 52, 57, 58). This reflects historical underfunding of Māori health research and the privileging of randomised controlled designs within academic publishing. From a kaupapa Māori perspective, this asymmetry does not indicate that Rongoā Māori is ‘unevidenced’, rather, it indicates that the evidence takes different forms and answers different questions.

Emerging research documents positive outcomes when Rongoā Māori is integrated with Western clinical practices (59). The Te Matahouroa trial placed a surgeon and a rongoā practitioner in simultaneous consultations with patients (n = 4). Although small, the trial resulted in high levels of satisfaction for both patients and rongoā practitioner, and significantly improved treatment adherence (e.g., smoking cessation, better medication management). The findings from this study suggest that bicultural collaboration creates a ‘safe cultural space’ that treats the whole person and their whānau rather than just the isolated physical ailment. One other study explored the relationship between Western surgeons and rongoā practitioners (60). The study found a ‘notable willingness’ among surgeons to integrate rongoā but identified that their understanding was ‘completely minimal’ or ‘limited at best’, often restricted to what they had learned in basic te reo Māori (Māori language) courses. Rongoā practitioners reported feeling overlooked by surgeons and that collaboration is currently ‘minimal’ because the non-Māori community has little knowledge of Rongoā Māori (60).

Experiential narratives from patients and tohunga detail successful outcomes using rongoā rakau (plant-based remedies), such as kawakawa (piper excelsum) and mamaku (cyathea medullaris) ointment for chronic skin conditions (dermatitis) after hospital prescribed medicines proved ineffective. Moreover, a recent review of rongoā rakau identified specific bioactive compounds in karamu (asperuloside), kūmarahou (quercetin, kaempferol, saponins, ellagic acid), and kawakawa (isovitexin/vitexin) that have documented anti-diabetic potential and effects on glucose metabolism (57, 61, 62). It suggests these plants could combat insulin resistance without the side effects (like weight gain or gastric distress) associated with conventional drugs like metformin. However, until recently, most claimed efficacies are not yet based on Western scientific methodologies, including modern laboratory testing or human clinical trials.

Service data from the Accident Compensation Corporation (ACC) no-fault injury insurance scheme, found that those receiving rongoā report significant improvement to short and long-term physical injury and emotional trauma. The benefits also extend to whānau (family/support network) through strengthened connections to cultural identity and practices. Of most significance, in the year 2023 to 2024, 41 per cent of those accessing rongoā via the ACC scheme identified as being of non-Māori descent (63). Since its introduction to ACC, rongoā services have significantly grown from 20 to 226 registered rongoā practitioners. By 2024, they had delivered 121,000 sessions to more than 12,000 individuals (64). Those findings suggest strong universal appetite for rongoā services.

Those findings are supported by usage data from NZ’s public health sector that indicates robust public trust in Rongoā Māori (65). A survey at Gisborne Hospital reported that 91% of inpatients had used traditional, complementary, or alternative therapies, the highest published rate in NZ. Within this cohort, Māori used Rongoā Māori four times more frequently than non-Māori, with over 90% of Māori women reporting its use. Notably, 97% of all respondents stated they would use the therapies within the hospital if available. However, Gisborne has the highest proportion of residents of Māori descent in all of NZ, according to data from the 2023 Census (66). Fifty-four per cent of the Gisborne population are of Māori whakapapa (genealogy), and 70.4 per cent of those under 25 in the region are Māori.

Meanwhile, new models continue to explore the potential for Rongoā Māori. The Tū Wairua project is currently exploring a decolonised approach to treating methamphetamine addiction using psilocybin-containing mushrooms within a marae-based (where formal meetings and discussions take place) rongoā framework (67). While physical therapies such as mirimiri (body work) has shown to support pain relief, stress reduction, mental clarity and body realignment (68).

We emphasise that no evidence for Rongoā Māori as a treatment method for EDs exists. However, our proposal for integration is justified by the totality of available evidence, qualitative, experiential, usage-based, and biological, combined with Te Tiriti Obligations and Māori treatment aspirations.

### Rongoā supported clinical management model

The argument for integrating Rongoā Māori with SSCM are based on its unique potential. Specialist Supportive Clinical Management provides a credible, evidenced based Western psychotherapy scaffold that, due to its core design, creates critical openings for Indigenous knowledge. However, when viewed through a rongoā lens, its limitations become clear, revealing why Rongoā Māori is not just complementary but essential for a complete therapeutic approach for Māori (see **Table 1**).

**Table 1:** Points of alignment: where SSCM and Rongoā Māori resonate.

Specialist supportive clinical management strength/component	Alignment with rongoā māori/kaupapa māori principles
Patient-led narrative.	Resonates with whakawhiti kōrero and the principle of following the client’s mana and self-determined healing path. It aligns with tino rangatiratanga (self-determination) in therapy.
Exploration of the self-perceived causal factors.	Creates the opening to explore causes rooted in whakapapa, whānau, or wairua.
Quality of life, option to explore life issues.	Mirrors the holistic intent of Te Whare Tapa Whā and rongoā, which never treat the tinana (body) in isolation from the hinengaro (mind), or wairua (spirit). Considers the intergenerational impact that treatment and rongoā will have across the whānau (family, support network). Parallels the rongoā goal of ora (wellbeing, vitality), which is the broader than mere symptom remission and encompasses living a full, engaged life.
Supportive, non-confrontational stance, strengths focussed and draws on past successes and their ideas.	Aligns with the manaaki (to care for, support) and aroha (compassion) that underpin the therapeutic relationships in rongoā.

Compared to other evidenced based treatments, SSCM is a relatively simple, flexible, non-prescriptive psychotherapy, with the exception of the clinical management focus on restoring healthy eating patterns and weight. Unlike rigid, theory- and protocol-driven therapies, SSCM’s supportive psychotherapy component is directed by patient’s own concerns and does not impose a specific model of causation or treatment. This creates a natural space for the client’s worldview, values, and self-perceived causal factors (e.g., trauma, whānau (family, support network) disruption, cultural dislocation) to take centre stage. Its dual focus seamlessly blends practical health clinical management (nutrition, weight focus) with supportive therapy exploration of patient concerns. This avoids the mind-body silo critiqued in Western medicine and acknowledges the physical restoration as interwoven with psychological wellbeing.

As a therapy, that requires ‘basic knowledge about ED and counselling,’ it is potentially more adaptable and teachable than highly specialised models. This flexibility is crucial for adapting its structure to hold different cultural content. Moreover, the evidence for SSCM has proven that it is equivalent to the “gold standard” therapies, thus providing a credible, evidence-based platform. This strategic legitimacy is important for implementation within mainstream health systems.

Before implementation, the following operational rules for Rongoā Māori must be defined (1): low-risk practices (karakia, mirimiri, whakawhiti kōrero) would be routinely available (2); rongoā rākau (plant remedies) would require case-by-case assessment, including consultation with rongoā practitioner (3); practices involving tapu would be respected but not replicated by non-Māori clinicians (4); conflicts between SSCM nutritional goals and rongoā practices would be resolved collaboratively with the patient, whānau (support network), and rongoā practitioner, prioritising clinical safety and cultural integrity.

Despite these alignments, SSCM remains a product of a Western paradigm. From a rongoā worldview, it is fundamentally partial and culturally decontextualised. Its limitations are not failures of design, but omissions of scope. **Table 2** illustrates how Rongoā Māori can be applied within the SSCM framework to ensure a culturally responsive treatment option.

**Table 2:** Limitations of SSCM from a Rongoā Māori perspective: the gaps requiring integration.

Gap identified from a rongoā perspective	What is missing and why it matters
SSCM provides structure (session time, supportive dialogue, clinical goals) however, from a rongoā perspective, it does not inherently include cultural-spiritual content.	Without the guiding cosmology of mātauranga Māori, the ‘patient-led’ exploration may never reach core wairua (spiritual) or ancestral whakapapa (genealogical) determinants of distress. The therapy risks being culturally shallow.
SSCM has no built-in processes to establish tapu/noa, acknowledge the mauri of the space or people, or open/close session in a spiritually conscious way (e.g., karakia or prayer, incantation). However, it is recommended that therapists in NZ delivery therapy in a culturally appropriate manner.	This can make the clinical space feel culturally unsafe for Māori, failing to honour the sacred dimension of healing (taha wairua) and potentially leaving spiritual unease unaddressed.
SSCM’s focus, even when supportive, remains on the individual’s psyche and behaviour.	This contradicts the foundational Māori view that identity and health are collective and relational. When appropriate, healing could involve whānau to address intergenerational patterns. SSCM lacks the tools for this.
SSCM may discuss ‘food,’ but it cannot conceptualise kai as a sacred connection to whenua (land) and atua (gods/deities). Nutritional advice is biochemical, not relational.	It misses a profound therapeutic avenue: healing through reconnection to Papatūānuku (mother earth) and other atua (gods/deities). It cannot incorporate rongoā rākau (plant remedies) or understand land and atua as a healer.
SSCM is not designed to explicitly address colonisation, land loss, or cultural alienation as pathogenic forces.	This ignores the lived realities for Māori including the ongoing impact of colonisation.
It validates knowledge from RCTs and clinical observation but has no way to value mahi matakite (insight), ancestral knowledge, or spiritual diagnosis.	This sustains epistemic injustice. The wisdom of a tohunga or a kaumātua (elder) is marginalised, and the client’s own culturally framed understanding of their illness may be subtly invalidated.
SSCM has no formal mechanism for resolving conflicts between Western and Māori healing approaches.	Collaborative protocols for shared decision-making when treatment recommendations differ.

## A shift in the overton window: weaving an integrated model

### Rāranga as the foundational metaphor

To illustrate the practical integration of Rongoā Māori and SSCM, we utilise the concept of rāranga or weaving. Weaving is a living art form passed down from tupuna (ancestors) and a strong symbol of the survival of Māori culture. Today, weaving skills are used to produce korowai (cloaks), and other practical objects such as kete (baskets), and whāriki (mats). Within this model, the patients’ therapeutic journey is understood as a whāriki in progress: each session adds a new thread, and the final pattern is unique to the individual.

*The whakataukī (proverb) grounding the model reinforces this principle:*

“Kotahi te aho ka whaiti, ki te kāpuia e kore e whati.”

“One strand of flax is easy to break, but many strands together will stay strong.”

In this context, strength emerges from the weaving of both systems, with the patient, Rongoā practitioner, and SSCM therapist each holding their own strand.

### The three realms of wellbeing

**Figure 2:**
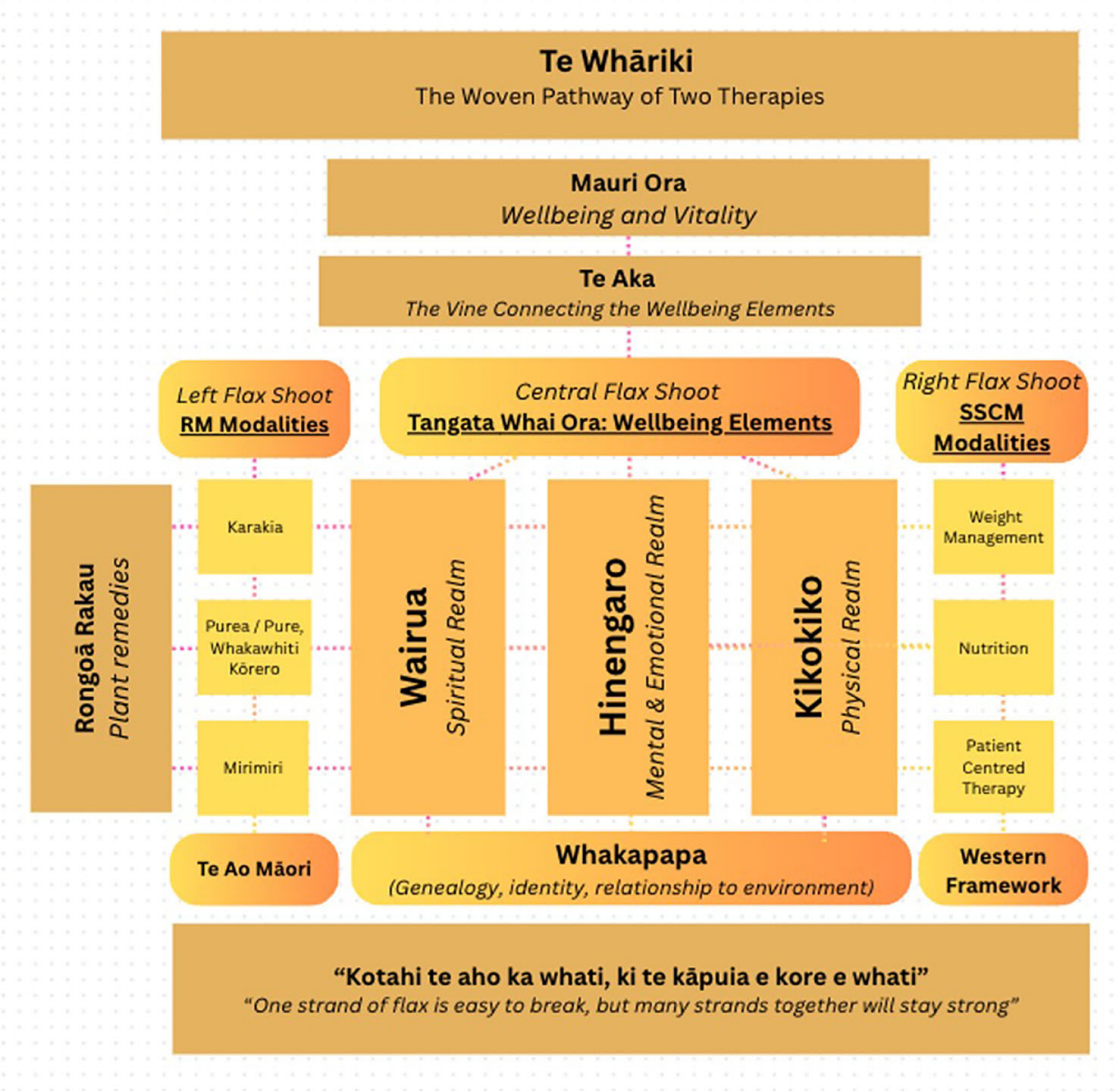
A visual representation of an integrated therapy model (RM-SSCM), conceptually grounded in the Māori practice of whāriki (weaving). In this model, the Te Aka vines symbolise the vital connective tissue between various elements of holistic wellbeing (wairua, hinengaro, kikokiko); when these elements are harmoniously aligned, the resulting state is one of wellbeing and vitality. The treatment methods of RM and SSCM and their specific modalities are positioned on either side of this central structure, offering complimentary pathways for care. Grounding the entire model is whakapapa, representing the experiences that inform a patient’s lived experience, illustrating that healing must begin with a deep understanding of one’s unique history and identity.

In **Figure 2**, three key elements for human wellbeing are identified: wairua (spiritual realm), hinengaro (real of the mind and emotions), and kikokiko (physical realm). Within the whāriki framework, these three elements form the vertical flax shoots and sit at the centre of the matt. They represent the person with disordered eating, in their totality. **Table 3** describes each element.

**Table 3:** Description of the central elements of wellbeing.

Central element	Realm	Description
Wairua	Spiritual realm.	Connection to spirituality, mauri (life force), and whakapapa
Hinengaro	Realm of the mind and emotions.	Thoughts, cognition, mental patterns, and emotional processing.
Kikokiko	Physical realm.	The body, including weight, nutrition, digestion, and somatic experience.

To the left of these central shoots sits the rongoā practitioner and their modalities. To the right, sits the therapist and the SSCM modalities. The patient moves between and across these strands, guiding which thread is pulled at which moment across their therapeutic journey.

### Whakapapa as the foundational realm of disordered eating

Beneath the whāriki, not as a vertical pillar but as the foundational ground, lies whakapapa (genealogy, ancestral connection, relational lineage). Whakapapa is the whenua (land) upon which the mat is woven. It encompasses whānau (support network), tūpuna (ancestors), hapū (sub-tribe), iwi (tribe), and the broader environment.

Within this model, disordered eating is understood to sit within the whakapapa realm. It is not primarily a pathology of the individual’s wairua (spiritual realm), hinengaro (realm of the mind and emotions), and kikokiko (physical realm), but a signal, an expression of imbalance, disruption, or rupture within the whakapapa field. These disruptions may include intergenerational trauma from colonisation (land alienation, or cultural suppression), grief from unresolved whānau loss (death, divorce, estrangement), disconnection from whenua (land), or cumulative exposure to various types of adversity.

The individual, in this framework, is not the source of the disorder but the carrier of a whakapapa imbalance. Disordered eating behaviours (restriction, bingeing, purging, excessive exercise) are understood as the body’s attempt to manage or express what cannot yet be resolved in the whakapapa realm.

Healing therefore requires addressing the whakapapa disruptions themselves, not merely managing the individuals’ symptoms. Both the rongoā practitioner and therapist attend to whakapapa, the rongoā practitioner through karakia (prayer, incantation), whakawhiti kōrero (talk therapy), and culturally specific practices; the SCCM therapist through patient-led dialogue on any life issues they wish to focus on. The two therapies are complementary: each addresses whakapapa in ways consistent with its own knowledge system, ensuring the patient’s genealogical and ancestral stories are held by both.

## The first session: weaving foundations through the Hui process

The first assessment would include the rongoā practitioner, SSCM therapist and person with disordered eating and their family (if desired). Framed within the hui process (69), a culturally responsive framework is used to enhance therapeutic alliance. The first session follows four steps (**Table 4**).

**Table 4:** The hui process in integrated session 1.

Step	Term	Function
1	Mihimihi	Welcome. The rongoā practitioner opens with karakia (prayer, incantation) and a brief speech welcoming everyone.
2	Whanaungatanga	Establishing connections. Each person present (patient, therapist, practitioner, and whānau) discloses their tribal and sub-tribal affiliations, work history, and their current roles or responsibilities related to the integrated therapy.
3	Kaupapa	Topic of the appointment. A summary of the integrated therapy is discussed, including benefits and risks, and initial assessment is undertaken. Any patient or whānau concerns are discussed.
4	Poroaki	Farewell. A summary of the session and next steps are offered to ensure the team are all on the same page.

The first session is about weaving strong foundations within a culturally safe environment. It establishes that both therapeutic systems are legitimate, that the patient’s whakapapa and whenua connections matter, and that the therapists will work in parallel with shared accountability.

## Ongoing therapy: parallel delivery with coordinated documentation

As the therapy continues, the rongoā practitioner or SSCM therapist will weave their different modalities based on the presenting needs of the patient. However, rongoā and SSCM therapies are undertaken in parallel, meaning the patient attends sessions with both, not joint sessions (beyond the initial assessment and periodic review hui, including the halfway point, and final session). Note-taking and sharing will ensure both the rongoā practitioner and SSCM therapist have absolute clarity on the patients’ progress, emerging needs, and shifting priorities.

This parallel structure preserves the integrity of each therapeutic system. The rongoā practitioner does not attempt to deliver SSCM, and the SSCM therapist does not attempt to deliver rongoā. Instead, coordination occurs through shared clinical notes, regular brief hui (peer related supervision) between the therapist and rongoā practitioner and joint reflection on the developing whāriki pattern, including how whakapapa is being addressed across both therapeutic strands.

## Clinical micro-level integration: illustrative examples

To illustrate how rongoā practices might be integrated at the clinical micro-level, we consider several examples, each understood through the whakapapa framework. We provide parallel examples from both therapeutic strands to demonstrate how whakapapa is held across the model.

Karakia (prayer, incantation) could be offered by the rongoā practitioner to the patient to recite before meals to reduce anxiety around food, creating a culturally grounded ritual for emotional regulation. Within this model, karakia before meals reconnects the individual to whakapapa, inviting ancestral support and restoring the mauri (life force) of kai. Anxiety around food is understood as a signal of whakapapa disruption, not merely a cognitive distortion to be managed through behavioural techniques alone. In parallel, the SSCM therapist might explore with the patient whether anxiety around food has appeared elsewhere in their whakapapa, asking, for example, “has anyone else in your whānau struggled with eating?” or exploring whānau attitudes to food, and body image, thereby holding whakapapa in clinical conversation while supporting nutritional stability.

Mirimiri (therapeutic message) could be offered by the rongoā practitioner to improve the body-mind connection through positive healing experience of the body. Mirimiri works with the kikokiko (physical body) but also opens whakapapa channels. Physical touch, when delivered with tikanga (cultural protocols), can release intergenerational trauma held in the body. Dissociation is reframed as a disconnect from whakapapa and the kikokiko realm, not merely a psychological defence mechanism. The SSCM therapist, aware of the mirimiri work through shared notes, might then support the patient to articulate in words what the body released, connecting the somatic experience to whakapapa narratives through the clinical management component of the therapy. In this way, the support arm of the therapy remains free, providing space for whatever the patient wants to talk about. This can enhance the patient’s capacity to ‘make sense’ of their experiences (what they see, feel, notice, sense etc), and to have conversations with their SSCM therapist about these. This can in turn help the patient to shift away from individualised ‘self-blaming’ pathology and toward an understanding of ED that feels more embodied and framed within its appropriate context (understanding from a cultural lens).

Whakawhiti kōrero (talk therapy within a rongoā framework) differs from standard SCCM dialogue by explicitly situating the patient’s story within whakapapa (genealogical) connections, and wairua (spirituality) rather than focusing solely on individual psychology. The rongoā practitioner asks not only “what happened to you?” but also “What happened in your whakapapa?” and “who are your ancestors?” The patient’s ED is explored as a possible carrier of unresolved ancestral grief, land loss, or intergenerational trauma. By contrast, in SSCM’s supportive psychotherapy component, whakapapa themes are not proactively invited. They may be noted during the assessment phase and addressed subsequently only if the patient raises them spontaneously. The difference, therefore, is not only how whakapapa is addressed, through cultural practice in rongoā versus clinical dialogue and narrative exploration in SSCM, but also whether it is proactively situated in the patient’s story from the outset.

These are illustrations only; specific practices would be determined through co-design with rongoā practitioners, clinicians, and Māori communities in future empirical work.

## Discussion

This conceptual analysis seeks to bridge the gap between Western and Indigenous wellbeing methodologies by proposing a rōngoa-informed adaptation of SSCM. We argue that prevailing treatment models have been a poor fit for Māori due to a narrow, culturally incongruent focus, contravening the obligations of Te Tiriti o Waitangi and UNDRIP (23, 28). The need to bridge traditional Māori and Western healing practices is necessary to address gaps in service delivery (57). In response, the newly developed ED glossary (He Papakupu: Ngā Māuiui Kai) and the holistic system of Rongoā Māori provide the necessary cultural and philosophical foundation. Specialist Supportive Clinical Management, with its patient-centred flexibility, simple pragmatic therapy approach to normalising eating and attaining or maintaining a healthy weight, without a predetermined causal or theoretical model, emerges as the most viable and adaptable Western psychotherapy for this cultural integration.

Our proposed integration reconceptualises health restoration as a process of rebalancing the realms central to wellbeing including wairua (spiritual), hinengaro (mind and emotions), and kikokiko (physical). It suggests that incorporating rongoā practices alongside SSCM’s structure can address the spiritual and relational dimensions of ED while supporting biomedical goals. This model represents a potential shift in the Overton Window of ED treatment, moving Indigenous healing from the margins to the centre of credible, effective care.

However, a strong theoretical case must be followed by evidence of acceptability, feasibility, effectiveness, and safety. The integration of any new component to an established therapy carries risk. Therefore, we present this not as a definitive solution, but as a necessary and promising pathway requiring systematic evaluation. Future steps must involve co-design with Māori communities, lived-experience experts, and rongoā practitioners to operationalise and safely pilot this model. Research must then employ kaupapa Māori methodologies to rigorously evaluate its feasibility, acceptability, efficacy and potential unintended consequences.

A critical window for this work has been opened by the national refresh strategy (4). If successfully developed and evaluated, the integration of Rongoā Māori and SSCM has the potential to form a complete bicultural therapeutic approach that honours Te Tiriti o Waitangi: SSCM upholding the Article 3: Right to Equity in health; and rongoā upholding Article 2 Rights to tino rangatiratanga (self-determination) over taonga (treasures), including health knowledge.

## Future directions: protocols for partnership and co-design

A Rongoā Māori informed approach to Specialist Supportive Clinical Management moves beyond cultural competence to cultural collaboration, offering a pathway to healing that addresses the biopsychosociocultural-spiritual determinants of health.

For this proposed transition from theory to ethical and effective practice, a critical next phase of work must focus on building structured protocols for collaboration between mainstream psychotherapy and rongoā practitioners. True integration requires more parallel practice; it necessitates a framework for respectful partnership that clarifies roles, communication pathways, and shared decision-making to safely and effectively co-support Māori experiencing EDs and their whānau. Future research must therefore prioritise the co-design of collaborative practice guidelines with rongoā practitioners and psychotherapists.

## Theoretical limitations

We acknowledge that the peer-reviewed literature on Rongoā Māori and Māori experiences of eating disorders is limited, a constraint that reflects not the absence of adequate rongoā knowledge, but rather the historical underfunding of Māori health research and the privileging of Western validation frameworks within academic publishing. From a kaupapa Māori lens, this limitation requires us to draw responsibly on diverse sources (qualitative, experiential, cultural) while being transparent about evidentiary differences. We do not claim equivalence between rongoā and Specialist Supportive Clinical Management evidence types but rather seek respectful synthesis across knowledge systems. We also acknowledge that our proposal remains theoretical. Empirical testing of feasibility, acceptability, and efficacy is required before any clinical recommendations can be made.

## Data Availability

The raw data supporting the conclusions of this article will be made available by the authors, without undue reservation.

## Footnotes

1 The Overton window refers to the range of topics that are politically palatable to the mainstream population at a specific time point.
